# Highly Selective Thiazole Derivatives as Promising Anti-*Toxoplasma gondii* Agents

**DOI:** 10.1021/acsomega.6c06743

**Published:** 2026-09-24

**Authors:** Ingrid de Oliveira Dias, Gabriel Cândido Moura, Lucas Manoel da Silva Sousa, Rafael Meyer Mariante, Ana Cristina Lima Leite, Juliana Quero Reimão

**Affiliations:** † 146840 Faculdade de Medicina de Jundiaí, Laboratory of Preclinical Assays and Research of Alternative Sources of Innovative Therapy for Toxoplasmosis and Other Sicknesses (PARASITTOS), Rua Francisco Telles 250, 13202-550 Jundiaí, Brazil; ‡ Federal University of Pernambuco, Department of Pharmaceutical Sciences, Avenida Artur de Sá S/N, 50740-520 Recife, Brazil; § 196605 Instituto Oswaldo Cruz, Laboratory of Structural Biology, Avenida Brasil 4365, 21040-360 Rio de Janeiro, Brazil

## Abstract

Toxoplasmosis, caused by the protozoan *Toxoplasma gondii*, remains a significant global health challenge, particularly for immunocompromised individuals. Current therapies, based on pyrimethamine and sulfadiazine, are limited by toxicity, suboptimal efficacy, and the emergence of resistant parasite strains, highlighting the need for safer and more effective alternatives. In this study, we evaluated the antiparasitic potential of six 2-pyridyl 1,3-thiazole derivatives—privileged scaffolds previously reported for potent antitumor activity but never investigated against *T. gondii*. In vitro assays were performed using intracellular tachyzoites of the RH strain and human foreskin fibroblasts to determine antiparasitic activity and host-cell cytotoxicity. The derivatives exhibited half-maximal effective concentrations (EC_50_) between 212 and 490 nM and cytotoxicity values (CC_50_) ranging from 1.5 to 48 μM. One compound, TAP-05, demonstrated a notable selectivity index of 183.26, exceeding that of the reference drug pyrimethamine, supporting its potential as a lead candidate for further optimization. Transmission electron microscopy of treated parasites revealed reduced ultrastructural signs of tachyzoite division and increased cytoplasmic vacuolization compared to untreated controls, along with occasional Golgi perturbations. These qualitative observations raise the hypothesis of a cytostatic effect of TAP-05 on parasite replication, which requires confirmation by dedicated functional assays. These findings identify a promising thiazole-based chemotype for toxoplasmosis drug discovery and warrant additional studies to elucidate mechanisms of action and assess in vivo efficacy.

## Graphical abstract

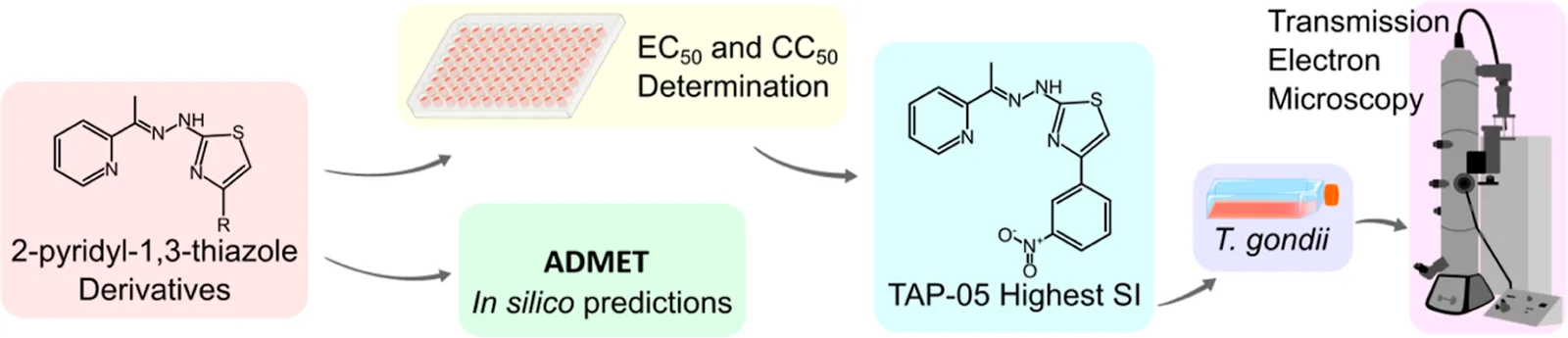

## Introduction

*Toxoplasma gondii* is an obligatory intracellular protozoan with a wide distribution, responsible for causing toxoplasmosis, the most prevalent zoonotic parasitic disease worldwide, affecting approximately 35% of the population over a lifetime.^1^ Infection occurs primarily through the ingestion of oocysts present in contaminated water or food and tissue cysts in raw or undercooked meat.^2^ Although most immunocompetent individuals remain asymptomatic, at-risk groups, such as pregnant women and immunocompromised patients, may develop severe manifestations.^3^ Congenital toxoplasmosis can lead to neurological and ocular complications in the fetus, while in immunosuppressed patients, such as those with HIV/AIDS or undergoing transplantation, parasite reactivation can result in cerebral toxoplasmosis, a severe and potentially fatal condition.^4^

Current treatments rely primarily on the combination of pyrimethamine and sulfadiazine, which, despite being effective against the active stage of the infection, have significant limitations, including high toxicity, adverse side effects, and limited efficacy against tissue cysts responsible for the chronic phase of the disease.^5^ Additionally, the need for prolonged treatment regimens and the potential for parasite resistance highlight the urgency for safer and more effective therapeutic alternatives.^6^

In medicinal chemistry, the concept of privileged structures refers to molecular scaffolds with affinity for multiple biological targets, making them promising starting points for drug development; these structures exhibit features that favor specific interactions with biomolecules, increasing the likelihood of desirable biological activity.^7^ Among privileged heterocyclic scaffolds, thiazole derivatives have attracted considerable attention because of their broad spectrum of biological activities, including antibacterial, antifungal, antiviral, anticancer, and antiparasitic effects.^8,9^ Although several thiazole-containing compounds have been reported to exhibit activity against protozoan parasites, including *T. gondii*, the pharmacological potential of individual thiazole subclasses varies considerably depending on their substitution pattern and molecular architecture.^8^

In this context, 2-pyridyl-1,3-thiazole derivatives represent a distinct and chemically versatile subclass that has been extensively explored for antitumor applications^10^ but remains largely unexplored as an antiparasitic scaffold. Previous studies demonstrated that these compounds exhibit potent antiproliferative activity against tumor cells and significant activity against *Trypanosoma cruzi*,^10,11^ indicating their ability to interfere with conserved cellular processes essential for cell survival. Furthermore, the presence of multiple positions amenable to chemical modification makes this scaffold particularly attractive for structure–activity relationship (SAR) studies and future lead optimization, representing an advantage for medicinal chemistry programs aimed at discovering new antiparasitic agents.

Given that *T. cruzi* and *T. gondii* are both intracellular protozoan pathogens that share analogous mechanisms of host cell invasion, intracellular survival, and immune evasion, we hypothesized that 2-pyridyl-1,3-thiazole derivatives previously shown to be active against *T. cruzi* may also exhibit activity against *T. gondii*.^11,12^ Moreover, the potential conservation of molecular targets between kinetoplastid and apicomplexan parasites supports the rationale for cross-species evaluation. This approach aligns with the strategy of identifying privileged scaffolds with broad antiparasitic potential rather than simply repurposing existing drugs.

Molina et al. evaluated the anti-*T. gondii* activity of a congeneric series of hydrazinyl-4-thiazolidinone esters, identifying the 2-pyridyl-4-thiazolidinone derivative 2B (Scheme 1) as a lead compound (EC_50_ = 0.20 μM, SI = 1000).^13^ Similarly, Lączkowski et al. investigated the antitumor and anti-*T. gondii* properties of 2,4-diaminotriazine-1,3-thiazole derivatives featuring substitutions at position 4 of the thiazole ring, revealing compound 4i as the most active antiparasitic agent in their series (EC_50_ = 30.7 μM, SI > 4.18).^14^ Based on these findings, we designed a novel series of 2-pyridyl-1,3-thiazole derivatives, TAP(01–06) (Scheme 1). This structural design was achieved through a bioisosteric replacement of the 4-thiazolidinone core in 2B with a 1,3-thiazole ring, combined with the incorporation of a 4-aryl moiety inspired by 4i (Scheme 1). Despite the promising pharmacological potential of these scaffolds, the anti-*T. gondii* activity of 2-pyridyl-1,3-thiazole derivatives remains largely unexplored. Given the limitations of current toxoplasmosis treatments, investigating these derivatives may not only identify new therapeutic candidates for the infection but also provide insights into potential antiparasitic mechanisms of action, paving the way for innovative strategies to combat the disease.

**Scheme 1:**
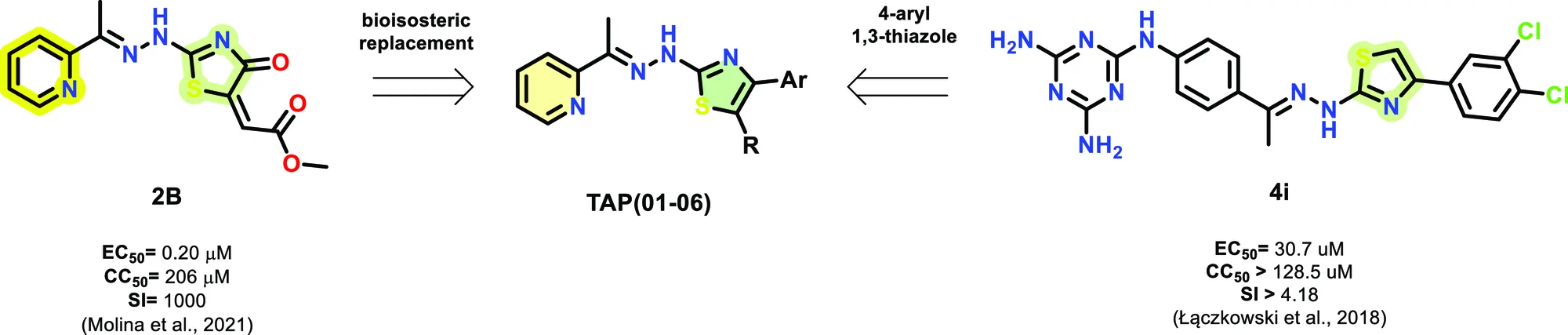
Structural Design Rationale for 2-Pyridyl-1,3-thiazole Compounds with anti-*Toxoplasma gondii* Activity

This study aims to evaluate the anti-*T. gondii* activity of 2-pyridyl-1,3-thiazole derivatives, exploring their potential as antiparasitic drug candidates. By investigating this previously unexplored chemotype, we seek to contribute to the development of safer and more effective therapeutic alternatives for toxoplasmosis.

## Results and Discussion

### In Vitro Activity against *T. gondii* and Host Cell Cytotoxicity

Compounds 2-(2-(pyridin-2-ylmethylene)­hydrazinyl)-4-phenyl-1,3-thiazole (TAP-01), 2-(2-(pyridin-2-ylmethylene)­hydrazinyl)-4-(4-tolyl)-1,3-thiazole (TAP-02), 2-(2-(pyridin-2-ylmethylene)­hydrazinyl)-4-(4-bromophenyl)-1,3-thiazole (TAP-03), 2-(2-(pyridin-2-ylmethylene)­hydrazinyl)-4-(4-fluorophenyl)-1,3-thiazole (TAP-04), 2-(2-(pyridin-2-ylmethylene)­hydrazinyl)-4-(3-nitrophenyl)-1,3-thiazole (TAP-05), 2-(2-(pyridin-2-ylmethylene)­hydrazinyl)-4-(naphthalen-1-yl)-1,3-thiazole (TAP-06) (Figure 1) were previously prepared and characterized as previously reported.^11^

**Figure 1:**
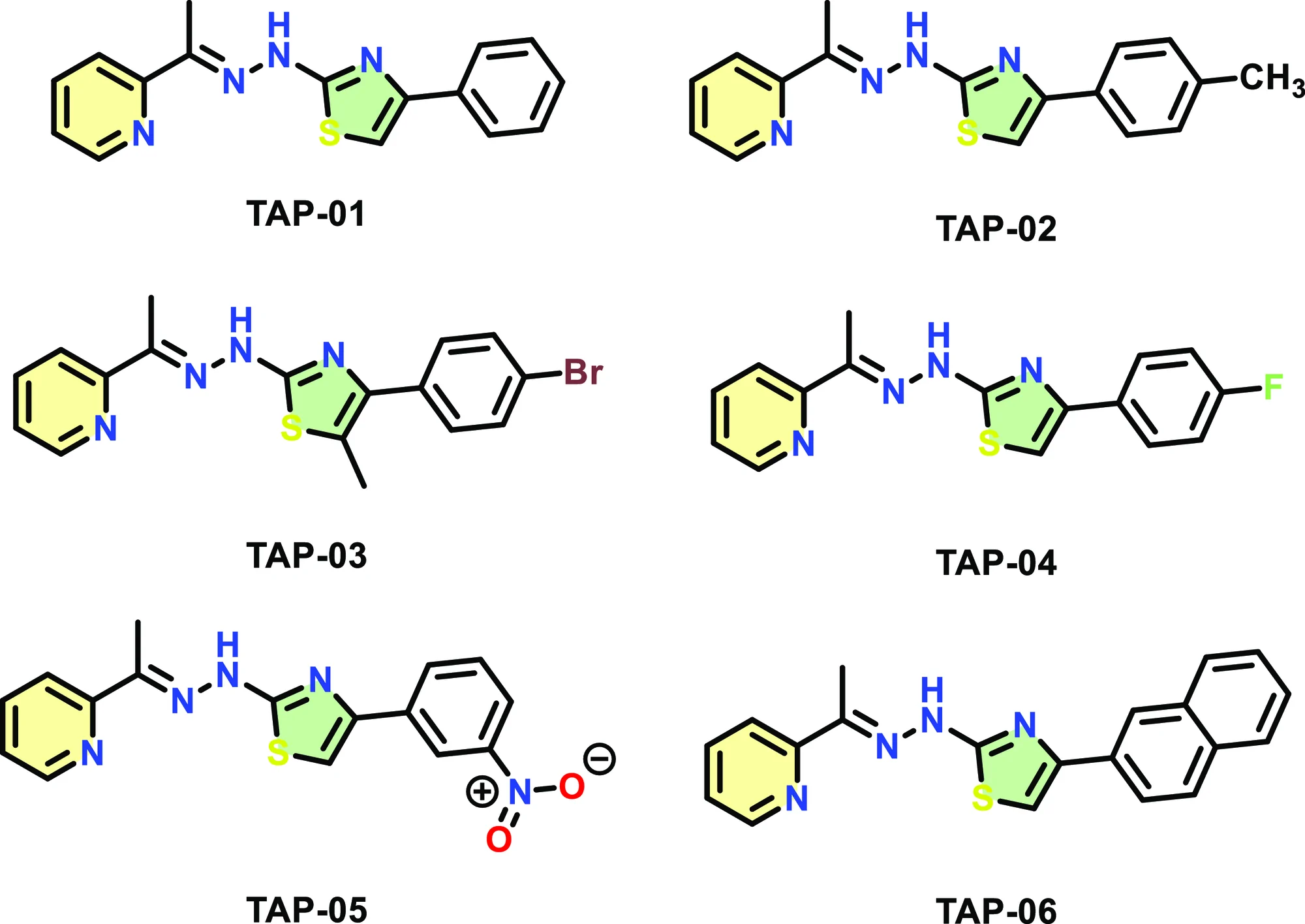
Structures of 2-pyridyl-1,3-thiazole derivatives.

The in vitro activity of 2-pyridyl-1,3-thiazole derivatives against *T. gondii* intracellular tachyzoites was assessed using the β-galactosidase assay, while cytotoxicity in human foreskin fibroblasts (HFF) was determined by the resazurin assay as previously described.^15^ The results are summarized in Table 1. Representative dose–response curves for the antiparasitic activity against *T. gondii* and cytotoxicity toward HFF cells are presented in the Supporting Information (Figure S1).

**Table 1:** Half Effective Concentration (EC_50_) against *Toxoplasma gondii* Intracellular Tachyzoites and Half Cytotoxic Concentration (CC_50_) against Human Foreskin Fibroblasts of 2-Pyridyl 1,3-Thiazole Derivatives and Pyrimethamine

compound	EC_50_ (μM) ± SD^a^	CC_50_ (μM) ± SD^b^	selectivity index^c^
TAP-01	0.279 ± 0.081	2.36 ± 0.758	8.44
TAP-02	0.355 ± 0.098	2.99 ± 0.055	8.44
TAP-03	0.212 ± 0.059	1.59 ± 0.039	7.50
TAP-04	0.490 ± 0.039	4.75 ± 2.107	9.69
TAP-05	0.264 ± 0.059	48.44 ± 3.739	183.26
TAP-06	0.216 ± 0.117	3.04 ± 0.034	14.06
pyrimethamine	0.381 ± 0.028	>50	>131

a EC_50_: half effective concentration against *T. gondii* intracellular tachyzoites, obtained by β-galactosidase assay.

b CC_50_: half cytotoxic concentration against human foreskin fibroblasts, obtained by Resazurin assay.

c Selectivity index: the ratio of CC_50_ in HFF to EC_50_ in intracellular tachyzoites; SD: standard deviation represents the mean of three independent experiments.

Among the tested compounds, TAP-03 exhibited the lowest EC_50_ value (0.212 ± 0.059 μM), indicating the highest potency against *T. gondii*. TAP-05 demonstrated the highest selectivity index (SI = 183.26), attributed to its relatively low EC_50_ (0.264 ± 0.059 μM) combined with a high CC_50_ (48.44 ± 3.739 μM), suggesting a favorable safety profile. TAP-06 also displayed a promising SI (14.06), with an EC_50_ of 0.216 ± 0.117 μM and a CC_50_ of 3.04 ± 0.034 μM.

Compared to pyrimethamine (EC_50_ = 0.381 ± 0.028 μM; CC_50_ > 50 μM; SI > 131), most derivatives exhibited lower EC_50_ values, suggesting higher potency. However, except for TAP-05, they generally exhibited lower SI values than pyrimethamine, mainly due to their moderate cytotoxicity. TAP-04 (SI = 9.69) and TAP-01/TAP-02 (SI = 8.44) demonstrated intermediate selectivity, while TAP-03 showed the lowest SI (7.50), indicating a more limited therapeutic window.

Overall, these findings highlight TAP-05 as the most selective derivative, presenting high antiparasitic activity with low cytotoxicity. Further investigations are warranted to investigate its mechanism of action and evaluate its potential as a lead candidate for toxoplasmosis treatment.

Although all derivatives displayed comparable submicromolar antiparasitic activity, their selectivity indices varied markedly, indicating that subtle structural modifications primarily influenced host-cell toxicity rather than parasite inhibition. In particular, TAP-05, bearing a 3-nitrophenyl substituent at the C4 position of the thiazole ring, exhibited a selectivity index approximately 13- to 24-fold higher than the remaining derivatives. Although the present compound set is too limited to establish definitive SAR, these findings suggest that the nature and position of the aromatic substituent substantially affect the balance between antiparasitic activity and mammalian cell cytotoxicity. This observation identifies the 3-nitrophenyl moiety as a promising structural feature for future lead optimization, which should be validated using a broader series of analogues.

Compounds TAP-01 through TAP-06 share a common pharmacophoric core comprising a 2-pyridyl ring, a hydrazone linker, and a 1,3-thiazole ring. They differ only in the aryl substituent at the C4 position of the thiazole, which modulates overall activity and selectivity. The enhanced selectivity of TAP-05 may arise from the distinctive electronic and physicochemical contributions of the *meta*-nitro substituent. In addition to providing two hydrogen-bond acceptors and strongly withdrawing electron density from the conjugated hydrazone-thiazole system, the nitro group is expected to improve the polarity-lipophilicity balance and preserve a favorable molecular conformation. Together, these effects could enhance parasite-target recognition while reducing nonspecific mammalian-cell toxicity.

The electronic influence of the nitro substituent is well documented in medicinal chemistry. As a potent electron-withdrawing group, the nitro moiety reduces electron density within the conjugated hydrazone–thiazole scaffold, thereby modulating the acidity of the hydrazone NH and increasing the electrophilic character of the molecule.^16^ Furthermore, such electronic perturbation is known to shift tautomeric equilibria and influence the conformational preferences of heterocyclic derivatives, which are critical for target-binding affinity in pharmacological systems.^17^

The biological activities observed for the 2-pyridyl-1,3-thiazole derivatives in this study reinforce the pharmacological versatility previously attributed to this chemical class. These scaffolds have been reported to display antiproliferative activity in tumor models and trypanocidal effects,^10–12^ suggesting their ability to modulate conserved cellular pathways across diverse biological systems. Such evidence provides a strong rationale for evaluating their potential against *T. gondii*, a parasite that depends on tightly regulated processes of replication, organelle biogenesis, and vesicular trafficking.

The in vitro results revealed a range of antiparasitic potencies among the six derivatives tested. TAP-03 showed the highest potency, exhibiting the lowest EC_50_ value, whereas TAP-05 combined potent anti-*T. gondii* activity with markedly reduced cytotoxicity, resulting in the highest selectivity index (SI) of the series. TAP-06 also inhibited parasite proliferation at low micromolar concentrations, but its cytotoxicity narrowed its therapeutic window relative to TAP-05. When compared to pyrimethamine, most derivatives demonstrated lower EC_50_ values, indicating strong intrinsic antiparasitic activity; however, their CC_50_ values were lower, which prevented them from surpassing the reference drug in selectivity. This pattern suggests that although the scaffold provides favorable antiparasitic potency, further optimization targeting host-cell tolerability will be necessary to generate candidates with improved therapeutic indices.

### In Silico ADMET Predictions

The ADMET (absorption, distribution, metabolism, excretion, and toxicity) properties of the entire series of 2-pyridyl 1,3-thiazole derivatives were evaluated through computational predictions using the SwissADME and Osiris Property Explorer platforms. Although TAP-05 emerged as the most promising lead based on its biological profile, evaluating all six derivatives allowed comparison of scaffold-dependent pharmacokinetic characteristics, identification of common medicinal chemistry liabilities, and generation of information to support future SAR studies and rational analogue optimization.

All tested compounds exhibited high gastrointestinal (GI) absorption, suggesting favorable oral bioavailability. However, none were predicted to cross the blood–brain barrier (BBB), indicating limited potential for central nervous system (CNS) penetration (Table 2). Additionally, none of the derivatives were identified as *P*-glycoprotein (*P*-gp) substrates, implying a lower risk of efflux-mediated drug resistance.

**Table 2:** ADMET Predictions Obtained Based on the Physicochemical Properties of the 2-Pyridyl 1,3-Thiazole Derivatives

pharmacokinetics^a^	TAP-01	TAP-02	TAP-03	TAP-04	TAP-05	TAP-06
GI absorption	high	high	high	high	high	high
BBB permeant	no	no	no	no	no	no
*P*-gp substrate	no	no	no	no	no	no
CYP1A2 inhibitor	yes	yes	yes	yes	yes	yes
CYP2C19 inhibitor	yes	yes	yes	yes	yes	yes
CYP2C9 inhibitor	yes	yes	yes	yes	yes	yes
CYP2D6 inhibitor	yes	yes	no	no	no	yes
CYP3A4 inhibitor	yes	yes	yes	yes	yes	yes
Log Kp	–5.22 cm/s	–5.05 cm/s	–5.01 cm/s	–5.26 cm/s	–5.62 cm/s	–4.64 cm/s

**druglikeness** ^a^	**TAP-01**	**TAP-02**	**TAP-03**	**TAP-04**	**TAP-05**	**TAP-06**
Lipinski	yes	yes	yes	yes	yes	yes
Ghose	yes	yes	yes	yes	yes	yes
Veber	yes	yes	yes	yes	yes	yes
Egan	yes	yes	yes	yes	yes	yes
Muegge	yes	yes	no	yes	yes	no

**medical chemistry** ^a^	**TAP-01**	**TAP-02**	**TAP-03**	**TAP-04**	**TAP-05**	**TAP-06**
PAINS	0 alert	0 alert	1 alert	0 alert	1 alert	0 alert
Brenk	1 alert	1 alert	1 alert	1 alert	3 alerts	1 alert
leadlikeness	no	no	no	no	no	no

**toxicity** ^b^	**TAP-01**	**TAP-02**	**TAP-03**	**TAP-04**	**TAP-05**	**TAP-06**
mutagenic	low	low	low	low	low	low
tumorigenic	high	high	high	high	high	high
irritant	no	no	no	no	no	no
reproductive effective	no	no	no	no	no	no

a Data obtained from Swiss ADME platform.

b Data obtained from OSIRIS platform.

Regarding metabolism, all compounds were predicted to inhibit multiple cytochrome P450 (CYP) isoforms, including CYP1A2, CYP2C19, CYP2C9, and CYP3A4, which may lead to potential drug–drug interactions. Notably, TAP-03, TAP-04, and TAP-05 were not predicted to inhibit CYP2D6, whereas the remaining compounds showed inhibitory potential for this enzyme. Skin permeation values (Log Kp) ranged from −5.62 to −4.64 cm/s, suggesting limited dermal absorption.

All derivatives met the criteria for Lipinski’s rule of five, Ghose, Veber, and Egan filters, supporting their potential as orally bioavailable drugs. However, TAP-03 and TAP-06 exhibited a single violation in the Muegge filter due to their high lipophilicity (XLOGP3 > 5). The bioavailability score was similar across the compounds (∼0.55), indicating a moderate probability of systemic absorption.

From a medicinal chemistry perspective, all derivatives presented at least one Brenk alert,^16^ primarily associated with imine groups, while TAP-03 and TAP-05 had additional structural alerts related to potential reactivity. TAP-03 also showed a PAINS alert, which may indicate a higher likelihood of assay interference. Lead-likeness assessment revealed violations in XLOGP3 (>3.5) for most derivatives, with TAP-03 also exceeding the molecular weight (*M*_W_ > 350) threshold. Synthetic accessibility scores ranged between 3.07 and 3.29, suggesting a relatively straightforward synthesis.

All compounds were classified as having low mutagenic potential and no reproductive toxicity or irritation risks. However, they were predicted to have a high tumorigenic risk, which warrants further experimental validation to assess their long-term safety profiles.

Overall, the computational analysis indicates that the favorable antiparasitic activity observed for this scaffold is accompanied by generally acceptable drug-likeness and oral absorption profiles. Importantly, because these predictions were generated for the entire compound series, they also reveal scaffold-wide liabilities, particularly CYP inhibition and predicted tumorigenicity, which should be addressed during future medicinal chemistry optimization. Thus, the ADMET evaluation complements the biological screening by providing guidance for the rational design of next-generation analogues rather than solely supporting the selection of TAP-05.

### Ultrastructural Imaging

HFF cells were infected with tachyzoites at a multiplicity of infection (MOI) of 1:1, and after 3 h, TAP-05 was added twice at its EC_50_ concentration. Following 48 h of treatment, the cells were washed, fixed, dehydrated, sectioned, and examined by transmission electron microscopy (TEM).

TEM revealed subtle ultrastructural alterations in *T. gondii*-infected HFF cells following 48 h exposure to the compound TAP-05. In untreated cells (Figure 2a–c), parasitophorous vacuoles (PVs) containing several tachyzoites in active division were observed, displaying well-preserved morphology and dense cytoplasmic organization. The surrounding host-cell cytoplasm showed intact mitochondria closely associated with the PV membrane.

**Figure 2:**
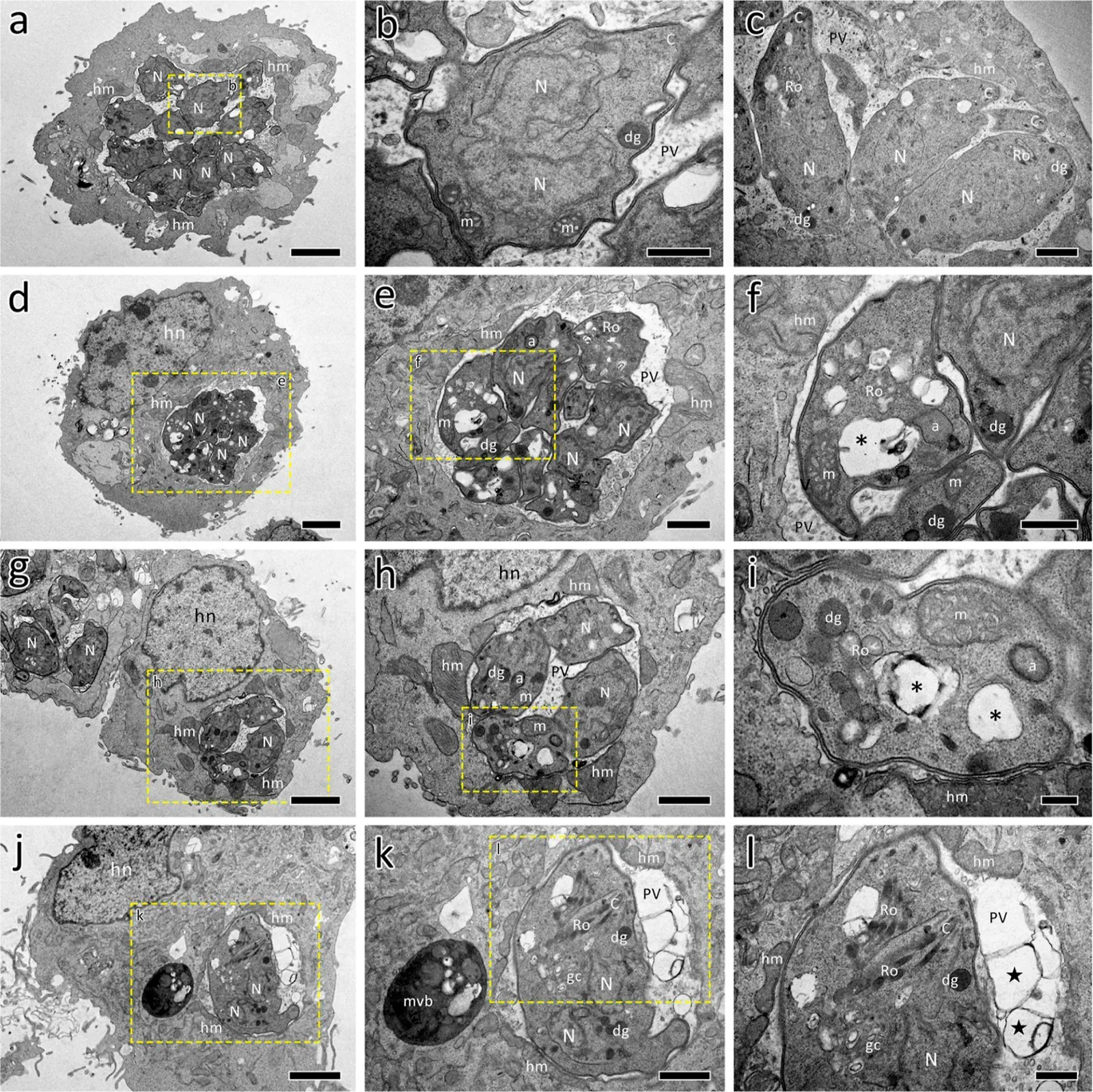
Transmission electron microscopy of *Toxoplasma gondii*-infected HFF cells after 48 h of treatment with TAP-05. [a–c] Untreated infected cells showing parasitophorous vacuoles (PVs) containing several tachyzoites in division, with preserved morphology and host-cell mitochondria (hm) surrounding the PV. [d–f] Cells treated for 48 h with TAP-05 showing a large PV with an individual tachyzoite displaying pronounced cytoplasmic vacuolization (asterisks). [g–i] Another treated cell showing PV containing a highly vacuolated tachyzoite. [j–l] Treated cell showing a PV with a single parasite exhibiting apparent division impairment and altered Golgi cisternae morphology (gc); the PV displays irregular “holes” resembling large vacuoles (stars). A multivesicular body-like structure (mvb) is present in the host-cell cytoplasm near the PV. N = nucleus; hn = host-cell nucleus; m = mitochondria; hm = host-cell mitochondria; a = apicoplast; Ro = rhoptries; dg = dense granules; C = conoid. Scale bars: 2.0 μm [a,d,g,j]; 1.0 μm [c,e,h,k]; 0.5 μm [b,i,l]; 0.2 μm [f].

HFF cells treated for 48 h with TAP-05 (Figure 2d–l) exhibited parasites showing structural alterations and a lower frequency of vacuoles containing dividing tachyzoites. In several vacuoles (Figure 2d–i), individual tachyzoites presented increased cytoplasmic vacuolization, while other vacuoles (Figure 2j–l) contained a single parasite with apparent defects in cell division and dilated or vesiculated Golgi cisternae. The PV in these fields also displayed irregular areas or “holes” resembling large intravacuolar spaces. In the same field, near the PV, a structure resembling a multivesicular body was noted in the host-cell cytoplasm, suggesting local cellular stress. Cytoplasmic vacuolization and reduced ultrastructural signs of active tachyzoite division were more frequently observed among TAP-05-treated parasites than in untreated controls raw counts provided in Supporting Information (Table S1); Golgi alterations were occasionally observed in treated samples, at a slightly higher frequency than in controls, a finding consistent with previous ultrastructural observations of Golgi cisternal disorganization induced by structurally related thiazole-pyridine hybrids in *Leishmania infantum*.^18^

Given the limited sampling depth intrinsic to ultrathin-section TEM and the comparative, nonrandomized screening strategy employed, these frequencies are reported descriptively and are not intended as statistically validated prevalence estimates. These qualitative findings, together with the absence of overt lytic changes and the preservation of most parasite organelles, are compatible with a cytostatic profile rather than rapid cytotoxicity. The enlarged intravacuolar spaces and host-cell multivesicular body-like structures may further reflect localized stress at the host–parasite interface. Confirming this hypothesis will require complementary functional assays quantifying parasite replication and secretory pathway activity.

Collectively, the biological, computational, and ultrastructural findings identify the 2-pyridyl-1,3-thiazole scaffold as a promising chemotype for anti-*T. gondii* drug discovery. While all six derivatives retained potent antiparasitic activity, TAP-05 clearly emerged as the lead compound owing to its markedly superior selectivity. Although the present study does not allow definitive SAR conclusions, the distinct performance of the 3-nitrophenyl derivative strongly suggests that appropriate modulation of the aromatic substituent can substantially improve the therapeutic window without compromising antiparasitic potency. Together with the ultrastructural evidence of a predominantly cytostatic effect, TAP-05 represents an attractive starting point for medicinal chemistry optimization. Future studies should focus on expanding the chemical series, establishing quantitative SAR, identifying the molecular target(s), and validating efficacy in animal models.

## Conclusions

This study identifies 2-pyridyl-1,3-thiazole derivatives, particularly TAP-05, as promising candidates for anti-*T. gondii* drug development. All six derivatives retained potent, submicromolar in vitro antiparasitic activity, while selectivity over host cells varied markedly with the substituent: most derivatives showed selectivity indices between 7.5 and 14, whereas TAP-05, bearing a 3-nitrophenyl substituent, reached a selectivity index of 183.26, surpassing the reference drug pyrimethamine, establishing it as the standout lead compound of the series. The ultrastructural findings are compatible with a cytostatic mode of action for TAP-05, a hypothesis that will require dedicated functional validation in future studies. These results establish the 2-pyridyl-1,3-thiazole core as a viable starting point for continued structure-guided optimization toward improved candidates for toxoplasmosis chemotherapy.

## Methods

### Compounds

Compounds 2-(2-(pyridin-2-ylmethylene)­hydrazinyl)-4-phenyl-1,3-thiazole (TAP-01), 2-(2-(pyridin-2-ylmethylene)­hydrazinyl)-4-(4-tolyl)-1,3-thiazole (TAP-02), 2-(2-(pyridin-2-ylmethylene)­hydrazinyl)-4-(4-bromophenyl)-1,3-thiazole (TAP-03), 2-(2-(pyridin-2-ylmethylene)­hydrazinyl)-4-(4-fluorophenyl)-1,3-thiazole (TAP-04), 2-(2-(pyridin-2-ylmethylene)­hydrazinyl)-4-(3-nitrophenyl)-1,3-thiazole (TAP-05), 2-(2-(pyridin-2-ylmethylene)­hydrazinyl)-4-(naphthalen-1-yl)-1,3-thiazole (TAP-06) (Figure 1) were previously prepared and characterized as previously reported.^11^

### Cell Culture Maintenance

*T. gondii* RH-2F1 tachyzoites (a transgenic clone expressing β-galactosidase)^19^ and human foreskin fibroblast (HFF) were maintained in Dulbecco’s Modified Eagle’s Medium (DMEM) without phenol red supplemented with 10% fetal bovine serum (FBS), l-glutamine, and gentamicin (D10) until a confluent monolayer was established. Once confluence was reached, the cells were washed, and the medium was replaced by DMEM containing 2% FBS, l-glutamine, and gentamicin (D2). Freshly egressed tachyzoites were then introduced into the culture, and following their subsequent egress, they were transferred to a new monolayer to maintain continuous propagation. All cultures were incubated at 37 °C in a humidified atmosphere with 5% CO_2_.^15^

### Half-Maximal Effective Concentration (EC_50_) Determination

To determine the EC_50_, HFF cells were seeded in 96-well plates with D10 at a concentration of 5 × 10^4^ cells per well and incubated at 37 °C in a CO_2_ incubator until reaching confluence (12–16 h). Subsequently, the wells were washed to remove nonadherent cells, and *T. gondii* tachyzoites (RH-2F1) in D2 medium were added at the same concentration. The plate was then incubated for 3 h to allow infection. After this period, compounds were added at an initial concentration of 1 μM, followed by serial dilutions from 1 μM to 15.62 nM per well, in duplicate, for 72 h at 37 °C in a CO_2_ incubator. β-Galactosidase activity was used to assess parasite viability as previously described.^15^ The following controls were included in the assay: infected HFF cell cultures without treatment (viability control), infected HFF cell cultures treated with 0.2% dimethyl sulfoxide (DMSO) (solvent control), infected HFF cell cultures treated with 1 μM Pyrimethamine (reference treatment control), and wells without cells (experimental blank).

### Half-Maximal Cytotoxicity Concentration (CC_50_) Determination

To determine the CC_50_, HFF cells were seeded in 96-well plates with D10 at a concentration of 5 × 10^4^ cells per well and incubated until reaching confluence (12–16 h) in a CO_2_ incubator at 37 °C. The compounds were then added at an initial concentration of 50 μM, followed by serial dilutions down to 0.78 μM, and incubated for 72 h under the same conditions. After this period, resazurin was added at a final concentration of 100 μM, and the plates were incubated for 4 h at 37 °C. Fluorescence readings were then performed using a Varioskan LUX plate reader (Thermo Scientific) with excitation at 565 nm and emission at 590 nm. The following controls were included in the assay: untreated HFF cell cultures (viability control), HFF cell cultures treated with 0.2% DMSO (solvent control), HFF cell cultures treated with 50 μM pyrimethamine (reference treatment control), and wells without cells (experimental blank). By integrating the obtained data, the Selectivity Index (SI) was calculated as the ratio of CC_50_ in HFF cells to EC_50_ in tachyzoites, providing a quantitative measure of a compound’s selective activity against the parasite relative to the host cell.^15^

### In Silico Predictions

In silico predictions of physicochemical and pharmacokinetic properties were performed to assess the drug-likeness and potential toxicity of the selected compounds. ADMET properties were evaluated using SwissADME, focusing on parameters such as gastrointestinal (GI) absorption and blood–brain barrier (BBB) permeability. Toxicity risks including mutagenicity, carcinogenicity, reproductive toxicity, and irritation potential, were predicted using Osiris Property Explorer.^15,21^

### Ultrastructural Analysis by Transmission Electron Microscopy

HFF cells were seeded in 25 cm^2^ culture flasks and maintained until confluence. They were then infected with tachyzoites at a multiplicity of infection (MOI) of 1:1. After 3 h, compound TAP-05 was added twice at its EC_50_ concentration. Following 48 h of treatment, cells were washed and fixed for 1 h with 2.5% glutaraldehyde in 0.1 M sodium cacodylate buffer (pH 7.2) containing 3.5% sucrose and 5 mM calcium chloride. After buffer washing, cells were postfixed for 20 min with 1% osmium tetroxide and 0.8% potassium ferricyanide, then scraped and transferred to conical tubes for acetone dehydration. Samples were embedded in EPON, sectioned using an ultramicrotome, stained with uranyl acetate and lead citrate, and analyzed using a Jeol 1011 (Jeol, Japan) transmission electron microscope at the Rudolph Barth Electron Microscopy Platform, Oswaldo Cruz Institute, Fiocruz. Three independent experiments were performed, each analyzing two grids per condition. Control grids were first surveyed to establish baseline morphology, and treated grids were then screened for deviations from it—an exploratory, nonrandomized strategy suited to identifying novel alterations but not to unbiased prevalence estimation. In total, 13 fields (15 parasitophorous vacuoles, 51 tachyzoites) were photographed in untreated controls, and 26 fields (39 vacuoles, 123 tachyzoites) in TAP-05-treated samples.

## Supplementary Material



## Notes

Figure S1. Dose–response curves of the six 2-pyridyl-1,3-thiazole derivatives against HFF (A to F) and *T. gondii* intracellular tachyzoites (G to L). Data are expressed as the percentage of cell viability relative to untreated controls. Representative results of three independent experiments Table S1. Frequency of ultrastructural alterations identified by transmission electron microscopy in *T. gondii* tachyzoites across three independent experiments. Frequency is reported descriptively; the nonrandomized, comparative screening strategy employed does not support unbiased prevalence estimation (see Methods) (PDF)
