# Supplementary material for: Highly Selective Thiazole Derivatives as Promising Anti-Toxoplasma gondii Agents
Source: ACS Omega. 2026 Sep 24;11(39):59807–14. doi: 10.1021/acsomega.6c06743 (PMC13647394; doi:10.1021/acsomega.6c06743)
Supplement: Supplementary file 1 [file ao6c06743_si_001.pdf]

Highly Selective Thiazole Derivatives as Promising Anti-*Toxoplasma gondii* Agents

Ingrid de Oliveira Dias[1], *Gabriel Cândido Moura*[1], *Lucas Manoel da Silva Sousa*[2], *Rafael Meyer Mariante*[3], *Ana Cristina Lima Leite*[2], *Juliana Quero Reimão*[1]*

[1]Faculdade de Medicina de Jundiaí, Laboratory of Preclinical Assays and Research of Alternative Sources of Innovative Therapy for Toxoplasmosis and Other Sicknesses (PARASITTOS), Rua Francisco Telles 250, 13202-550, Jundiaí, Brazil.

[2]Federal University of Pernambuco, Department of Pharmaceutical Sciences, Avenida Artur de Sá S/N, 50740-520, Recife, Brazil.

[3]Instituto Oswaldo Cruz, Laboratory of Structural Biology, Avenida Brasil 4365, 21040-360, Rio de Janeiro, Brazil.

*corresponding author e-mail: julianareimao@g.fmj.br

Supporting Information

Table S1. Frequency of ultrastructural alterations identified by transmission electron microscopy in *T. gondii* tachyzoites across three independent experiments. Frequency is reported descriptively; the non-randomized, comparative screening strategy employed does not support unbiased prevalence estimation (see Methods).

	**Control (n = 51 tachyzoites, 15 vacuoles)**	**TAP-05-treated (n = 123 tachyzoites, 39 vacuoles)**
Cytoplasmic vacuolization	15 (29.4%)	66 (53.7%)
Golgi alterations	4 (7.8%)	13 (10.6%)
Signs of active division	8 (15.7%)	1 (0.8%)

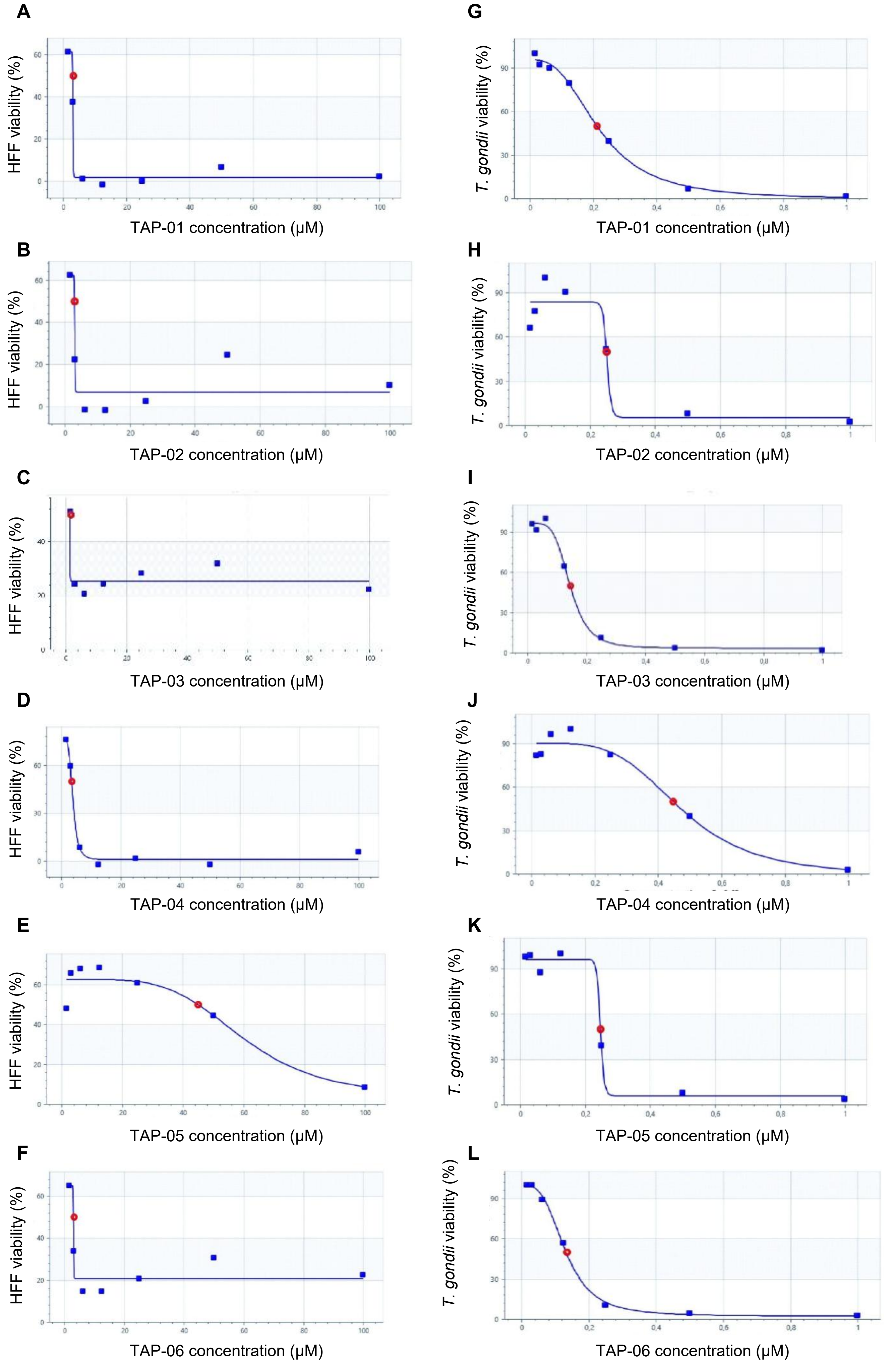

Figure S1. Dose–response curves of the six 2-pyridyl-1,3-thiazole derivatives against HFF (A to F) and *T. gondii* intracellular tachyzoites (G to L). Data are expressed as the percentage of cell viability relative to untreated controls. Representative results of three independent experiments.
